# Idiopathic Iliopsoas Muscle Hematoma

**DOI:** 10.31662/jmaj.2023-0178

**Published:** 2024-02-09

**Authors:** Shinya Otsubo, Junki Mizumoto

**Affiliations:** 1 Center for Medical Training, Ehime Seikyo Hospital, Ehime, Japan; 2 Department of Medical Education Studies, International Research Center for Medical Education, Graduate School of Medicine, The University of Tokyo, Tokyo, Japan

**Keywords:** Emergency Medicine, Hematoma, Iliopsoas muscle

A 77-year-old man complained of severe right lateral back pain. It had developed acutely nine days earlier when the patient was riding bicycle on a steep incline; the pain relieved seven days earlier. After cycling again on the day before presentation, the pain recurred. On the day of the presentation, the pain suddenly worsened.

He had to lay down on the bed and could not move his lower limbs; he remained afebrile. Computed tomography revealed distended right iliopsoas muscle (Figure 1). There was no contrast enhancement within the lump or along its periphery, reducing the likelihood of an iliopsoas abscess. Considering the homogeneously contrasted lump and abrupt onset of pain, idiopathic iliopsoas hematoma was ascertained to be the most likely diagnosis. Laboratory tests revealed no coagulopathies. Shrinkage of the iliopsoas lump was confirmed on the fourth day after the admission (Figure 2), concomitant with pain relief, leading to a definitive diagnosis.

**Figure 1.**
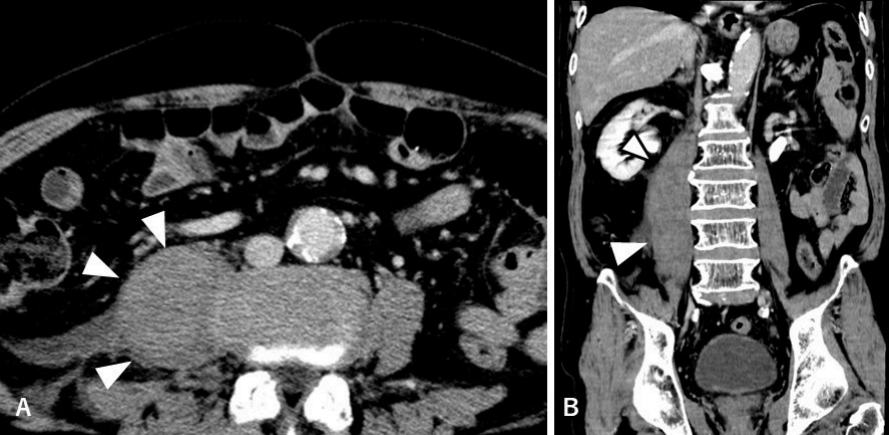
Swollen right iliopsoas muscle (white arrowheads) on contrast CT scan. (A) Horizontal view. (B) Coronal view.

**Figure 2.**
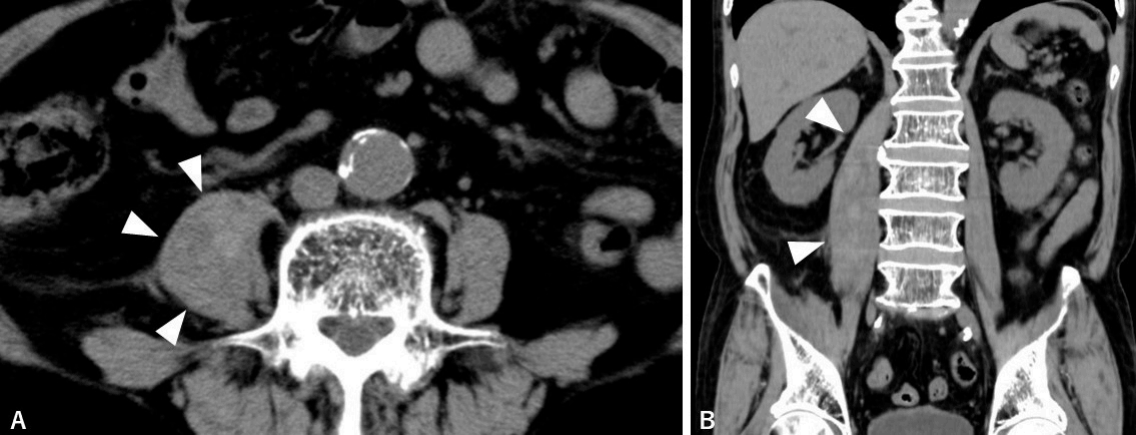
Shrunk hematoma (white arrowheads) on non-contrast CT on fourth day. (A) Horizontal view. (B) Coronal view.

This case highlights that iliopsoas hematomas can develop in patients without predisposing factors ^(1)^, and even moderate mechanical stress can trigger their development ^(2)^. Furthermore, the pain progressing in a stepwise manner suggests a potential link between repetitive strain and hematoma development.

## Article Information

### Conflicts of Interest

None

### Author Contributions

Concept: S.O., J.M.; design: S.O., J.M.; data collection or processing: S.O., J.M.; analysis or interpretation: S.O., J.M.; literature review: S.O., J.M.; and writing: S.O., J.M.

### Informed Consent

Informed consent was obtained from the patient.

### ORCID iD

Shinya Otsubo: 0009-0007-0689-653X

Junki Mizumoto: 0000-0002-0783-7351
